# An In Vitro Evaluation of the Antimicrobial Activity of Probiotics Against Endodontic Pathogens

**DOI:** 10.7759/cureus.26455

**Published:** 2022-06-30

**Authors:** Garapati Venkata Charan Teja, Mahali Raghu Nandana Raju, Uppu Lavanya Neelima Reddy, Uppalapati V V Satyanarayana, Devatha Praneeth, Kumpatla Maheswari

**Affiliations:** 1 Department of Conservative Dentistry and Endodontics, Lenora Institute of Dental Sciences, Rajahmundry, IND; 2 Department of Conservative Dentistry and Endodontics, Meghna Institute of Dental Sciences, Nizamabad, IND

**Keywords:** e. faecalis, in vitro, candida albicans, endodontic pathogens, probiotics

## Abstract

Background and aim: Despite scientific evidence that even some microorganisms may be useful, endodontic intervention has persisted to prioritize the removal of all microorganisms from the root canal system. Indeed, information regarding the significant role of probiotic microorganisms in endodontic treatment has been sparse. This study aimed to carry out an in vitro evaluation of the antimicrobial activity of probiotics against endodontic pathogens.

Methods: The evaluation was carried out in three stages. In Stage 1, the agar cup well procedure was used to analyse the efficiency of probiotics microorganisms against* Enterococcus faecalis* bacteria and *Candida albicans *microorganisms in the planktonic stage. In Stage 2, a deferred antagonistic experiment was used to determine the activity of probiotic microorganisms against endodontic pathogens like *E. faecalis* and *C. albicans* in the planktonic phase. In Stage 3, biofilm phase evaluation of an intracanal probiotic microorganism carrier was done. The region of maximum inhibition was measured at the end of Stages 1 and 2. The antimicrobial activity was recognized when the dimension of the region of maximum inhibition was 10 mm or above. The colony-forming unit/millilitre was measured at the end of Stage 3.

Results: There was marked antimicrobial activity of probiotic microorganisms against the pathogenic microorganisms *E. faecalis* as well as *C. albicans* in Stages 1 and 3, i.e., during the evaluation involving agar cup and evaluation at the biofilm stage. However, no antimicrobial activity of probiotic microorganisms was observed against pathogenic endodontic microorganisms in Stage 2, i.e., during evaluation involving the use of the deferred antagonistic technique.

Conclusion: It can be concluded that probiotic therapy is a promising antibacterial treatment approach that should be further investigated. This study shows that probiotics can help effectively in endodontic treatment and that more in vitro as well as in vivo research is needed to fully appreciate the advantages of bacteriotherapy in the field of endodontics.

## Introduction

The goal of endodontic therapy is to complete healing of periapical periodontitis, and the success of therapy is usually measured in eradicating the actual infection and preventing re-infection. It's worth noting that Kakehashi et al. found that study participants having no bacteria did not experience inflammation of periodontal tissues at the root apex despite physical access of the pulp of their molars to the oropharynx. However, control samples with typical oral microbiota acquired considerable radiolucency at the area around the root apex [1]. The flora of the root canal includes different types of microorganisms, with anaerobic species predominating. The root canal microenvironment is ideal for anaerobic microorganisms, which can degrade accessible amino acids and polypeptides to meet their metabolic requirements. Following infection, many microbial species interact among themselves, causing microbial population changes [2].

Furthermore, such microbial relationships are involved in the formation of complex microbiota in endodontic settings as well as ecological management. As a result, there is an ongoing debate in the field of endodontics about how much we can truly eradicate pathogenic germs from root canals. Multiple auxiliary and lateral canals were discovered during microscopic investigations of serial slices of the roots of various teeth [3]. The most favourable result that can be accomplished is a diminution in the biological burden on these branches, which are never fully free of microorganisms. Most likely, the effectiveness of endodontic treatments is due to a decrease in the number of germs, the removal of the most inflamed or dead tissue, and a healthy systemic background.

The assumption that all germs should always be eliminated from the root canal space, regardless of virulence or other qualities, limits traditional assumptions [4]. However, extensive studies in the disciplines of bacteriology as well as probiotics have led to the suggestion that the microbial community should be kept in a dynamic equilibrium. The microbial community is defined as the typical microbiological component of all people and animals that is essential for good health maintenance. When given in sufficient doses, probiotics are a group of living microorganisms that provide a physiological range of benefits to the general health of hosts [5].

In this approach, the normal bacterial species of the oropharynx can be referred to as the "oral microbial community," and probiotics can be injected into tooth root canals to improve the oral microbial resilience [6]. Immune modification, reducing the impact of inflammatory processes, formation of strong substances against microorganisms such as peroxide substances, carboxylic acids, as well as antimicrobial peptides, synthesis of mucin, suppression of epithelial incursion by hindering pathogenic organisms, mucosal conformance, activation of immunoglobulin A, as well as contestability with certain other microbiota, which would include harmful microorganisms, are all mechanisms by which probiotic work [7]. Probiotics appear to create a biofilm in the oral cavity that defends oral mucosa from pathogenic bacteria by occupying an area that might emerge as a subsequent pathogen habitat. Probiotics have already been used in dentistry to avoid dental caries and treat mouth and gut-related halitosis, candidiasis of the oropharynx, and diseases of the periodontium [8].

Notwithstanding, there seems to be limited research towards the utilisation of probiotic microorganisms for intracanal health, and probiotic microorganisms haven't been investigated in contexts of which variants are impactful or what colony-forming unit (CFU)/millilitre count is required to eliminate pathogens. According to a study, *Lactobacillus* exhibited no suppressive activity against *Enterococcus faecalis* [9]. Commonly available probiotics in the market were found to be efficient against endodontic infections, despite the fact that most preparations comprised low numbers of live organisms and provided minimal medical effect at the time of acquisition. Even with better-defined isolates, study results can be different because of how products are made and how stable they are [10,11].

In view of the influence of probiotic microorganisms in endodontic treatment, there is little unanimity among studies. These investigations also did not use the antagonistic method to assess the antibacterial capabilities of normal flora. As a result, this study was conducted to see if certain probiotics are efficacious in the management of endodontic infection. We hypothesised that when evaluated in an in vitro system, probiotic microorganisms could remove or reduce the number of *E. faecalis* bacteria and *Candida albicans* fungi in their planktonic as well as biofilm microbiological stages.

## Materials and methods

Research methodology and selection of probiotic variants and pathogenic variants

To test our theory, we devised an in vitro experiment based on the study findings of Seifelnasr [11]. Probiotics' anti-microbial performance was evaluated in the planktonic stage and biofilm stage. Isolated colonies of *Lactobacillus plantarum* having strain specifications of American Type Culture Collection (ATCC) 8014, strains of *Lactobacillus rhamnosus* having a specification of ATCC 7469, and strains of *Bifidobacterium bifidum *having specifications of ATCC 11863, purchased from the HiMedia center located in Mumbai (India), were chosen based on a literature study that demonstrated their efficacy as probiotics. Endodontic pathogens like *E. faecalis* bacteria with strain specifications of ATCC 29212 and *C. albicans* strains with strain specifications of ATCC 10231 were chosen and bought from HiMedia.

Stage 1

By utilising the agar cup well procedure, we analysed the efficiency of probiotic microorganisms against *Enterococcus* bacteria and *C. albicans *yeast in the planktonic stage.

*L. plantarum *and *L. rhamnosus* colonies with an optical density (OD) of 0.1 at a 620-nm wavelength were transplanted onto approximately 20 ml of sterilised melted agar and cooled to 45±2°C. This combination was properly mixed before being placed into a sterilized, unfilled Petri plate to set. Purified specimens of the *Lactobacillus* species were propagated in a 100-ml volumetric flask comprising De Man, Rogosa agar broth, and special MRS (De Man, Rogosa and Sharpe) agar broth having a pH of 6.0 and subsequently incubated at 37°C for 72 hours in mini-aerotolerant conditions to produce a cell-deprived precipitate (CDP) and cell-deprived filtrate product (CDFP). The precipitate comprised unrefined bacteriocin, and the technique of centrifugation was used to obtain a cell-deprived solution.

Cell-free supernatant (CFS) was then screened through a 22-micron filter membrane and tested for antimicrobial properties. For evaluation, unprocessed CFS, CFS adapted to a pH of 6.0 with the help of 1N sodium hydroxide, and unprocessed CFS mixed in a ratio of 1:2 were the three forms of CFS used. A sterilised steel cork digger was used to create circular reservoirs having a diameter of 10 mm in each of the discs. One hundred fifty of the *Lactobacillus* probiotic specimens were carefully administered in these reservoirs. The discs were incubated at a temperature of 37°C for 24 hours in an aerobic environment. The region of maximum inhibition (RMI) was assessed after incubation. *B. bifidum* was grown for 48 hours in glucose-rich broth in anaerobic environments at 37°C. The density of the cultures was subsequently regulated to 2 McFarland equivalents. At 37°C, experimental pathogenic microorganisms were grown in glucose-rich broth for 20 hours.

Stage 2

In this stage, we used a deferred antagonistic experiment to find out how probiotic microorganisms affect endodontic pathogens like *E. faecalis* and *C. albicans* in the planktonic phase.

*Lactobacilli *were calibrated to an optical density value of 0.1 at a 600-nm wavelength and seeded in 1-cm streaks in trypticase soy yeast isolate calcium agar (TSYCa) with the help of a sterilised cotton swab. The TSYCa plate contains 1.5% bacterial culture agar, 2% yeast extract, 0.25% calcium carbonate (CaCO_3_) agar, and 0.25% tryptic soy broth (TSB). Under microaerophilic conditions, the culture was incubated for 24 hours at 37°C. All TSYCa plates containing probiotic strains as well as pathogenic strains were cultured in an aerobic environment for 24 hours at 37°C. All experimental steps were carried out three times. With the help of a cotton swab, pure pathogenic microorganism cultures were smeared perpendicularly to the lines of probiotic microorganism cultures.

The suppressive action of probiotics was judged substantially positive in both stages of planktonic stage examination if the RMI produced by the probiotic microorganisms against the pathogenic microorganisms was at least 10 mm or more.

Stage 3

Here, we did biofilm phase evaluation of an intracanal probiotic microorganism carrier.

Poloxamer F 127 (407) was mixed for 10-15 minutes with a magnetic stirrer in cool MRS broth at a concentration of 30% until a homogeneous mixture was obtained. It was sterilised before being stored at 4°C until testing. The following were the guidelines for the control testing: Collections of *E. faecalis* and *C. albicans* were made in TSB to an optical density of 0.25, whereas collections of *L. plantarum*, *L. rhamnosus*, and *B. bifidum* were made in TSB to an optical density of 0.3-0.45.

Using an ultraviolet (UV) spectrophotometer, all equities were calibrated to the confluence at a 600-nm wavelength. Then, 9 ml of poloxamer was mixed with 500 ml of pathogenic colonies inside a test tube, and mixed thoroughly at 4°C in a refrigerator to ensure a homogeneous mixture. This combination was then incubated in aerobic conditions (at 37°C for 48 hours) prior to serial dilutions of pathological microorganisms containing biofilm specimens being formed and deposited on the brain heart infusion (BHI) agar medium to quantify colony-forming units (CFUs) of bacteria.

Statistical analysis

The data was analysed using independent sample t-tests, chi-square tests, and binary logistic regression. A convergence difficulty developed when we attempted to use a binomial regression analysis to estimate the antimicrobial activity of probiotic microorganisms against *C. albicans* and *E. faecalis*. To calculate overall risk ratios (RRs) and their 95% confidence intervals (CIs), a simplified Poisson regression method was developed. All analyses were performed employing Stata software, version 14 (StataCorp, College Station, TX).

## Results

Stage 1: Analysis involving the agar cup/agar well method

The RMI was 20.81±0.594 mm when strains of *L. plantarum *were investigated against pathogenic *E. faecalis *bacteria. The RMI was found out to be 20.51±0.627 mm when strains of *L. rhamnosus* were investigated against pathogenic *E. faecalis* bacteria. When strains of *B. bifidum* were investigated against pathogenic *E. faecalis*,* *the RMI was found to be 19.31±0.533 mm. These data reflect the antimicrobial activity of strains of *L. plantarum* probiotics, *L. rhamnosus* probiotics, and *B. bifidum* probiotics (Table 1).

**Table 1:** Details about the region of maximum inhibition produced by probiotics against pathogenic Enterococcus faecalis SD: standard deviation; SE: standard error; CI: confidence interval

	Region of maximum Inhibition				Data of descriptions			
	n	Mean values	SD values	SE values	95% CI for mean values		Minimum limit	Maximum limit
					Lower bound values	Upper bound values		
*Lactobacillus plantarum* probiotic microorganisms	25	20.81	0.594	0.264	20.46	21.16	20	21
*Lactobacillus rhamnosus* probiotic microorganisms	25	20.51	0.627	0.274	20.14	20.88	20	21
*Bifidobacterium bifidum* probiotic microorganisms	25	19.31	0.533	0.244	18.81	19.61	19	20

On carrying out analysis of variance (ANOVA) to analyse the variation between the groups and within the groups, it was found that the variation among the groups and within the groups was statistically significant (p=0.001) (Table 2).

**Table 2:** Analysis of variance for region of maximum inhibition by probiotic microorganisms against Enterococcus faecalis df: degrees of freedom

	Analysis of variance				
	Sum of squares values	df values	Mean square values	F value	p value
Between-groups comparison	23.711	3	7.411	38.996	0.001
Within-group comparison	7.211	38	1.337		
Total	30.922	31			

The RMI was 21.11±1.778 mm when strains of *L. plantarum* were investigated against the pathogenic *C. albicans* species in Stage 1 analysis involving the agar well method. The RMI was found out to be 21.11±1.778 mm when strains of *L. rhamnosus* were investigated against the pathogenic *C. albicans* species. When strains of *B. bifidum *were investigated against pathogenic *E. faecalis *bacteria, the RMI was 20.41±1.594 mm. These data reflect the antimicrobial activity of strains of *L. plantarum*, *L. rhamnosus*, and *B. bifidum *probiotics against *C. albicans* (Table 3).

**Table 3:** Details about the region of maximum inhibition produced by probiotics against Candida albicans SD: standard deviation; SE: standard error; CI: confidence interval

	Region of maximum inhibition				Data with descriptions			
	n	Mean values	SD values	SE values	95% CI for mean values		Minimum limit	Maximum limit
					Lower bound values	Upper bound values		
*Lactobacillus plantarum* probiotic microorganisms	25	21.11	1.778	0.322	20.63	21.59	20	21
*Lactobacillus rhamnosus* probiotic microorganisms	25	21.11	1.778	0.322	19.63	20.59	20	21
*Bifidobacterium bifidum* probiotic microorganisms	25	20.41	1.594	0.264	18.06	19.76	19	20

On carrying out ANOVA to analyse the variation between the groups and within the groups, it was found that the variation among the groups and within the groups was statistically significant (p=0.034) (Table 4).

**Table 4:** Analysis of variance for the region of maximum inhibition by probiotic microorganisms against Candida albicans df: degrees of freedom

	Analysis of variance				
	Sum of squares values	df	Mean square values	F value	p value
Between-groups data	4.378	3	2.744	5.477	0.034
Within-group data	11.211	28	1.485		
Total	4.478	30			

Stage 2: Analysis involving deferred antagonistic technique

Any of the probiotic bacteria had little antibacterial effect against any of the endodontic pathological microorganisms.

Stage 3: Evaluation at the biofilm stage

The CFU/ml of *E. faecalis* microorganisms reduced to 5.96 x 10^7^ when it was investigated against strains of *L. plantarum *probiotic microorganisms with 89.89% reduction in growth. The result was statistically significant (p=0.001). When *E. faecalis* microorganisms were investigated against strains of *L. rhamnosus* probiotics, the CFU/ml of *E. faecalis* reduced to 8.55 × 10^7^ with 90.60% reduction in growth. The result was statistically significant (p=0.004). The CFU/ml of *E. faecalis* microorganisms reduced to 6.1 × 108 when it was investigated against strains of *L. plantarum *probiotic microorganisms with 35.46% reduction in growth. The result was statistically significant (p=0.001) (Table 5).

**Table 5:** The CFU/ml and reduction in the growth of Enterococcus faecalis at the biofilm stage when treated against probiotic microorganisms CFU: colony-forming unit

	CFU/ml	Reduction in growth	p value
*Lactobacillus plantarum* probiotic microorganisms	5.96 × 10^7^	89.89%	0.001
*Lactobacillus rhamnosus *probiotic microorganisms	8.55 × 10^7^	90.60%	0.004
*Bifidobacterium bifidum *probiotic microorganisms	6.1 × 10^8^	35.46%	0.001

The CFU/ml of *C. albicans* microorganisms reduced to 2.46 × 10^7^ when it was investigated against strains of *L. plantarum* probiotic microorganisms with 42.06% reduction in growth. The result was statistically significant (p=0.001). When *C. albicans* microorganisms were investigated against strains of *L. rhamnosus* probiotics, the CFU/ml of the *C. albicans *microorganism reduced to 4.79 × 10^6^ with 91.80% reduction in growth. The result was statistically significant (p=0.004). There was no antimicrobial activity observed when *C. albicans* was investigated against *B. bifidum *probiotic microorganisms (Table 6).

**Table 6:** The CFU/ml and reduction in the growth of Candida albicans at the biofilm stage when treated against probiotic microorganisms CFU: colony-forming unit

	CFU/ml	Reduction in growth	p value
*Lactobacillus plantarum* probiotic microorganisms	2.46 × 10^7^	42.06%	0.001
*Lactobacillus rhamnosus *probiotic microorganisms	4.79 × 10^6^	91.80%	0.004
*Bifidobacterium bifidum* probiotic microorganisms	-	-	-

There was marked antimicrobial activity of probiotic microorganisms against the pathogenic microorganisms *E. faecalis* as well as *C. albicans* in Stages 1 and 3, i.e., during the evaluation involving agar cup and at the biofilm stage. However, no antimicrobial activity of probiotic microorganisms was observed against pathogenic endodontic microorganisms in Stage 2, i.e., during the evaluation involving the use of deferred antagonistic technique.

## Discussion

Despite indications that even some microorganisms may be useful, endodontic intervention has persisted to prioritize the removal of all microorganisms from the root canal system. Indeed, information regarding the significant role of probiotic microorganisms has been sparse. *Lactobacillus *and *Bifidobacterium* are the most frequently utilised probiotic microorganisms [11]. We investigated the feasibility of employing such species in endodontic therapy to promote bacteriotherapy. Initial illnesses of the decaying dental pulp usually have a mixed flora, with anaerobic type Gram-negative bacteria predominance. Conversely, anaerobic type Gram-positive bacterial species like *E. faecalis *and *C. albicans* yeast are more common in chronic infection. *C. albicans* is the most commonly detected yeast from root obturated teeth with periodontitis in the area around the root apex due to its biphasic character, which permits it as being the ubiquitous co-agglomerate in biofilms [12]. With its proton pump activities, *E. faecalis* tends to be very impervious to calcium hydroxide treatments, allowing it to persist in seclusion and develop a biofilm. The current in vitro study included components that focused on development (Stages 1 and 2) and implementation (Stage 3). Pure cultured isolates of probiotic microorganisms and endodontic pathological microorganisms were utilised both in planktonic and biofilm stages to eliminate bias [13].

In this study, there was a marked antimicrobial activity of probiotic microorganisms against the pathogenic microorganisms *E. faecalis* as well as *C. albicans* in Stages 1 and 3, i.e., during evaluation involving agar cup and at biofilm stage. However, no antimicrobial activity of probiotic microorganisms was observed against pathogenic endodontic microorganisms in Stage 2, i.e., during evaluation involving the use of deferred antagonistic technique [14].

Intense studies into probiotics continue to uncover new advantages arising from these microbes. Probiotics are taken in a direct or an indirect manner through a variety of food types, demonstrating their broad range of beneficial effects on humans. For a very long time, probiotics were primarily used to maintain gut health by avoiding or treating infections, modifying the host immunological response, and increasing vitamin secretion, among other things. Researchers from all over the world have recently become interested in the possible benefits of using probiotic microorganisms in the domain of oral health. Recent research, including clinical trials, clearly suggests that probiotics can help prevent and treat oral infections, such as tooth decay and other periodontal diseases. Probiotics have the unique capacity to form a biofilm, which functions as a protective lining and aids in the replacement of any pathogen that thrives in biofilms [15,16].

When tested, the antibacterial activities of 15 different *Lactobacillus*, *Bifidobacterium*, *Lactococcus*, *Streptococcus*, and *Bacillus* strains versus Gram-positive and Gram-negative harmful bacteria demonstrated that putative probiotics have the ability to suppress specific infections. The formation of organic compounds from lactose fermentation and the resulting reduction of culture pH could be one of the key regulatory mechanisms. Once the spectrum of organic acids was examined, it was discovered that lactic acid and acetic acid were the most important final products of probiotic-associated metabolism [17,18].

Strains' haemolytic reactivity and sensitivity to some of the most frequently used antimicrobial compounds, both of which are regarded basic characteristics, were also investigated. The antibacterial effect was primarily genus specific, but there were also considerable variances between species. Researchers found that children who were given the *L. rhamnosus* SP1 variant in milk for a duration of 10 months had a reduced burden of the carious teeth, a lower percentage of cavitated lesions, and a reduced occurrence of fresh lesions [19]. The *Streptococcus salivarius* M18 strain containing probiotics in the form of tablets was tested for three months in kids with an elevated danger of dental caries [20,21]. The general incidence of dental caries was significantly decreased. Similar findings were reported after three months of dairy intake with the *L. paracasei* SD1 variant. The probiotic strain was found to colonise the oral cavity of subjects in as early as three months, and then gradually declined to extremely low levels by 12 months. Altogether, the findings show that the daily use of probiotic *L. paracasei* SD1 strain may be suggested in children who are at an elevated risk of caries to reduce the number of *Streptococcus mutans* accountable for the early phases of dental decay [22].

The agar cup well and postponed antagonism procedures were used in Stages 1 and 2, respectively. The most pragmatic approach for testing antibiotic susceptibility is the agar cup/agar well technique, which is used in most workplaces. Both probiotic microorganism groups (*Lactobacillus* and *Bifidobacterium* strains) showed antimicrobial properties against *E. faecalis* and *C. albicans* specimens in their planktonic forms in an initial in vitro research. During Stage 2 of the investigation, the antimicrobial properties of probiotic microorganisms was tested once more using the antagonism testing. The antibacterial capabilities of typical nasopharyngeal flora were assessed using the delayed antagonistic approach. It identifies bacterial products with suppressive properties using a bacteriocin-like suppressive compound. Probiotic microorganisms, however, seemed to have no impact on endodontic pathogenic microorganisms in this study's antagonistic test [23,24].

Stage 3 of the trial was the implementation stage, in which we evaluated the efficiency of an innovative probiotic carrier system in the root canal space. This approach combined 30% poloxamer with a probiotic-rich MRS agar broth. Poloxamers comprise non-ionic, biodegradable polyethylene oxide with polypropylene oxide polycaprolactone that are employed as surfactants, emulsifiers, solubilizing agents, dispersion agents, and in vivo absorption amplifiers in pharmaceutical preparations.

These practical ingredients have advantages due to inverse thermosensitivity, which allows them to be dissolvable in water at low temperatures (typically 4°C) and convert into gel at elevated temperatures. These characteristics make them promising delivery carriers for intracanal therapies in between clinical visits [25].

Colony densities (CFU/ml) demonstrated significant growth constraints for *E. faecalis *bacteria and *C. albicans* yeast using the evaluated probiotic microorganisms in this investigation. This exploratory in vitro investigation highlighted the potential advantages of diverting attention from the necessity for total pathogen clearance to the reinstatement of normal human microbiome. Probiotics can prevent the production of endodontic pathogens in diseased root canals, allowing good bacteria to flourish and reestablish pulpal health [26].

It is possible to formulate a new two-step treatment protocol. Cleaning, shaping, and irrigation of the root canal, and activation may be used on the initial visit to reduce the bacterial concentration and eliminate biological tissues. The poloxamer-based probiotic combination could then be introduced into the space of the root canal and then left for about a week. Following disinfection, obturation could be performed on the second visit [27]. If organisms survive the procedure, the existence of probiotic microorganisms inside the space of root canals may create settings that are more favorable for successful endodontic treatment. Furthermore, we anticipate that probiotics might be combined with poloxamer to be used as a root canal sealant. In the future, we recommend that endodontic bacteriotherapy should concentrate on bringing helpful bacteria into the root canal space while removing only pathogenic flora, rather than attempting to achieve the unattainable objective of a sterile canal [28].

There were some limitations of this research. It was an in vitro study that did not correspond to the normal environment of the oral cavity. Therefore, more in vivo research should be carried out in the natural environment to acknowledge the results in a better manner.

## Conclusions

Probiotics have an important place in various applications in the dental field. It is a novel method to provide good bacteria to the area where there are infective pathogens, thus helping in limiting the spread of the disease. It is possible to draw the conclusion that probiotic therapy is a potentially useful antibacterial treatment strategy that needs to be researched further. This study demonstrates that the use of probiotics can be a successful component of endodontic treatment; nevertheless, further in vitro and in vivo research is required to have a complete understanding of the benefits of bacteriotherapy in the practice of endodontics.
